# Loss-of-Imprinting of HM13 Leads to Poor Prognosis in Clear Cell Renal Cell Carcinoma

**DOI:** 10.3390/biom14080936

**Published:** 2024-08-02

**Authors:** Floris Voorthuijzen, Cedric Stroobandt, Wim Van Criekinge, Tine Goovaerts, Tim De Meyer

**Affiliations:** 1 Department Data Analysis and Mathematical Modelling, BIOBIX Lab of Bioinformatics and Computational Genomics, Ghent University, Coupure Links 653, B9000 Ghent, Belgium; floris.voorthuijzen@ugent.be (F.V.); cedric.stroobandt@ugent.be (C.S.); wim.vancriekinge@ugent.be (W.V.C.); tine.goovaerts@ugent.be (T.G.); 2 Cancer Research Institute Ghent (CRIG), Ghent University, C. Heymanslaan 10, Ingang 36—Verdieping 1, B9000 Ghent, Belgium; 3 Bioinformatics Institute Ghent—Nucleotides 2 Networks (BIG N2N), Ghent University, Technologiepark Zwijnaarde 71, B9052 Zwijnaarde, Belgium

**Keywords:** KIRC/ccRCC, loss-of-imprinting (LOI), *HM13*, *H13*, *CRYBB2P1*

## Abstract

Genomic imprinting refers to the epigenetic silencing of one of both alleles in a parent-of-origin-specific manner, particularly in genes regulating growth and development. Impaired genomic imprinting leading to the activation of the silenced allele, also called canonical loss-of-imprinting (LOI), is considered an early factor in oncogenesis. As LOI studies in clear cell renal cell carcinoma (ccRCC) are limited to *IGF2*, we performed a genome-wide analysis in 128 kidney normal solid tissue and 240 stage 1 ccRCC samples (TCGA RNA-seq data) to screen for canonical LOI in early oncogenesis. In ccRCC, we observed LOI (adj. *p* = 2.74 × 10^−3^) of *HM13* (Histocompatibility Minor 13), a signal peptide peptidase involved in epitope generation. *HM13* LOI samples featured *HM13* overexpression, both compared to normal solid tissues (*p* = 3.00 × 10^−7^) and non-LOI (*p* = 1.27 × 10^−2^) samples. Upon adjustment for age and sex, *HM13* expression was significantly associated with poor survival (*p* = 7.10 × 10^−5^). Moreover, *HM13* overexpression consistently exacerbated with increasing tumor stage (*p* = 2.90 × 10^−8^). For *IGF2*, LOI was observed in normal solid tissues, but the prevalence did not increase in cancer. In conclusion, *HM13* LOI is an early event in ccRCC, causing overexpression leading to poor prognosis.

## 1. Background

Clear cell renal cell carcinoma (ccRCC/KIRC) accounts for 2–3% of cancers worldwide and about 92% of all renal cancers [1,2]. The prevalence of ccRCC is the highest in Western countries, where it is the ninth most common neoplasm, and the global incidence is projected to increase when more countries adopt a Western lifestyle [3]. When detected early, the 5-year survival rate can be as high as 93%, yet upon metastasis, it drastically drops to only 12% [3]. Therefore, adequate screening and subtyping are paramount to successful treatment [4]. Similarly, prognostic markers may be used to stratify patients according to risk, which is relevant for treatment management [5]. Here, we focused on imprinting deregulation in ccRCC as a potential source of clinically relevant biomarkers. 

Genomic imprinting refers to parent-of-origin-specific gene silencing, where solely the maternal or the paternal allele is expressed. This phenomenon is regulated by epigenetics, and imprinted genes are typically involved in growth regulation. Epigenomic alterations can deregulate this mechanism, which may result in loss-of-imprinting (LOI), i.e., re-expression of the normally silenced allele. LOI of growth-promoting genes has hence been associated with early tumorigenesis [6]. For example, insulin-like growth factor 2 (*IGF2*) is a paternally expressed imprinted gene on chromosome 11, and LOI was demonstrated in ccRCC and several other tumor types [7,8,9,10]. Importantly, even in healthy individuals, LOI of *IGF2* is not uncommon and occurs with aging, yet it predicts cancer risk [11,12] and was—in a murine prostate cancer model—found to be sufficient to increase the rate of neoplastic development on its own [13]. No clear evidence for LOI of other genes than *IGF2* in ccRCC has been reported so far.

The revolution in large-scale omics data generation has made bioinformatics an important contributor to oncology research. Large databases such as The Cancer Genome Atlas (TCGA) offer a goldmine of (epi)genomic, transcriptomic, proteomic, clinical, and other types of data in normal and tumor tissues, which can be integrated into comprehensive cancer-specific or pan-cancer analyses [14]. As LOI research in ccRCC has been virtually completely limited to *IGF2*, here, we rely on the large-scale TCGA dataset to perform a genome-wide screen for LOI in ccRCC and to evaluate the clinical implications.

## 2. Materials and Methods

### 2.1. Samples

Kidney data were obtained from TCGA for 128 solid tissue normal (STN) kidney samples (all tumor-adjacent samples obtained from KIRC (72), KIRP (kidney renal papillary cell carcinoma, 32), and KICH (kidney chromophobe, 24) substudies) and 530 KIRC tumor samples (all deduplicated: in case of duplicates, the sample with the most recent timestamp was retained). Tumor samples which upon histological inspection [15] were found not to be actual ccRCC were removed from the dataset, resulting in 482 ccRCC tumor samples. For (loss-of-) imprinting analysis, only the 240 stage 1 ccRCC tumor samples were considered. Called copy number variation (CNV; GISTIC scores), mutation, and Infinium HumanMethylation450k data were downloaded from MEXPRESS (v2019, Ghent, Belgium), which relied on Xena to obtain TCGA data [16]. These data were similarly filtered to only retain the confirmed ccRCC samples [15].

### 2.2. Imprinting Detection

KIRC solid tissue normal and primary tumor RNA-seq data were downloaded in the BAM (GRCh38) format from TCGA through the GDC portal (v24.0, Bethesda, Maryland, USA). Imprinting detection and associated data preprocessing were performed as described in Goovaerts et al. [17]. The methodology focuses on monoallelic expression, as imprinting manifests itself as either the paternal or the maternal allele being consistently (and typically completely) silenced across samples. In summary, after estimating allele frequencies from raw allele counts, a Hardy–Weinberg Equilibrium (HWE)–conform binomial mixture model is constructed, now also adjusting for “inbreeding” (coefficient of 0.0103667, estimated as in [18]). In this model, the heterozygous distribution is split up according to the degree-of-imprinting parameter i. An i equal to 0 corresponds to no imprinting and a single peak of heterozygous samples, whereas an i of 1 corresponds to complete imprinting with two heterozygous peaks undistinguishable from the homozygous ones. For statistical testing, the estimated î can be compared against the null hypothesis (i = 0) by means of a likelihood ratio test (FDR of 0.05). Further filters used in the pipeline are minimal median coverage > 4, #samples > 30, initial minor allele frequency (allele counts) > 0.1, minor allele frequency (genotyping estimate) > 0.15, P symmetry < 0.05, GOF > 0.80, minimal effect size î > 0.60, and robust median imprinting > 0.80 (see Goovaerts et al. [17] for an in-depth description of these parameters). To avoid including non-imprinted loci due to artefacts, we additionally filtered on a gene-level FDR (based on geometric mean of all SNP-level *p*-values per gene) of 0.05 and the presence of at least two significantly imprinted SNPs in the gene. As the latter step removed a few known loci very likely to be imprinted—*NHERF* Family PDZ Scaffold Protein 2 (*SLC9A3R2*); Paternally Expressed 10 (*PEG10*); Necdin (*NDN*); *GNAS* Complex Locus (*GNAS*); Rabphilin 3A Like (*RPH3AL*)—we manually added these loci to the table of imprinted genes.

### 2.3. Loss-of-Imprinting, Differential Expression, DNA Methylation, Copy Number Variation, and Mutation Analyses 

In line with Goovaerts et al. [17], loss-of-imprinting (LOI) is defined as imprinted loci (in normal solid tissues) featuring re-expression of the silenced allele in cases. However, this methodology was updated to reflect the integer nature of the allelic count data, i.e., LOI was detected by considering the least (most) expressed allele per sample as success (failure) and performing binomial logistic regression to compare the degree of success (i.e., re-expression) between both populations. This reflects that every sample’s “degree of LOI” can be defined as the read coverage of its least over its most expressed allele (1 for perfect heterozygotes, 0 for both perfect homozygotes). Hence, for each imprinted SNP this per-sample ratio can be modeled as a function of control–case status via one-sided binomial logistic regression, with a significant and positive case–control regression coefficient (i.e., more success = more biallelic expression) indicating LOI in stage 1 ccRCC. These LOI analyses were performed for all SNPs detected to be imprinted—as some genes may feature non-imprinted SNPs due to transcript-specific imprinting [17]—and the geometric mean was used to obtain a consensus LOI *p*-value over imprinted SNPs.

Canonical LOI refers to the combination of LOI and expression upregulation in cases [17]. For gene-level differential expression analysis, expression data were downloaded from MEXPRESS (v2019, Ghent, Belgium), which relied on Xena to obtain TCGA data [16], and analyzed via R’s t.test function on log(RPKM + 1). For genes with missing expression data in Xena/Mexpress, DE results were obtained at the SNP level using the reference- and variant-allele count, via R’s *t*-test function on library size-corrected log count data (marked ^$^ in Table 1), after which the *p*-values were combined into gene-level results by the geometric mean. To evaluate the impact of LOI on expression, stage 1 LOI samples were first identified as those stage 1 tumor samples featuring a least expressed allele count of at least 2 and a least expressed allele fraction (over total count over both alleles) of at least 10%. Subsequently, paired DE analysis via R’s *t*-test function, on log(RPKM + 1), was performed between stage 1 ccRCC LOI/non-LOI and their matched solid tissue normal kidney samples. Mutation (N = 336) and copy number variation (N = 234) data were also obtained from MEXPRESS (v2019, Ghent, Belgium). Furthermore, *t*-tests were performed on CNV data to measure the relation between CNV and expression, and chi-squared tests to measure the relation between CNV and LOI. In the methylation analysis (N tumor = 297, N STN = 160), beta values were transformed into M values on which limma was performed to test for differential methylation between the stage 1 LOI tumor, stage 1 non-LOI tumor, and solid tissue normal samples, and between the tumor and solid tissue normal samples across all stages. The results were transformed back to beta values for ease of interpretation.

### 2.4. Clinical Analyses

Cox regression was performed for the genes showing canonical LOI. The analyses were performed across all tumor stages, adjusting for age, sex, and tumor stage. Gene expression was included in the model as a continuous variable. For graphical representation, samples were grouped above or below tumor mean expression, and Kaplan–Meier graphs were plotted using these groups. ANOVA (on log(RPKM + 1)) was performed to determine the relationship between canonical LOI gene expression and tumor progression.

## 3. Results

First, we used an updated version of the (loss-of-) imprinting detection methodology described by Goovaerts et al. on 128 kidney solid tissue normal (STN, all tumor-adjacent) and 240 KIRC stage 1 case sample RNA-seq data from The Cancer Genome Atlas (TCGA) (see Section 2.2 and Section 2.3 for details). We focused on stage 1 cancer, as we aimed at detecting LOI relevant for early carcinogenesis and tumor progression rather than LOI caused by later-stage cancer. In our design, stage 1 ccRCC and STN samples did not significantly differ with respect to the potential major confounders including age (STN: 60.77 +/− 13.28 years old, cases: 60.00 +/− 12.88, independent samples *t*-test *p* = 0.41, F-test for equality of variance *p* = 0.68), and sex (STN: 33% female; case: 41% female; chi-squared *t*-test *p* = 0.17). 

The imprinting detection analysis in STN kidney yielded twenty-one candidate imprinted genes (Table 1), including nineteen genes previously identified to be (putatively) imprinted—such as Histocompatibility Minor 13 (*HM13*); *H19* Imprinted Maternally Expressed Transcript (*H19*); Maternally Expressed 3 (*MEG3*); and Mesoderm Specific Transcript (*MEST*)—but also two novel candidate genes without prior evidence, Crystallin Beta B2 Pseudogene 1 (*CRYBB2P1*) and Lethal Giant Larvae Homolog 2 (*LLGL2*). Note that these candidates may also exhibit random monoallelic expression (RME) or other allele-specific effects, given that also the Major Histocompatibility Complex, Class II, DQ Alpha 2 (*HLA-DQA2*)—known to feature RME—was detected by our pipeline. *IGF2* SNPs did not meet our predetermined imprinting thresholds, and *IGF2*-specific results are further discussed below.

**Figure 1:**
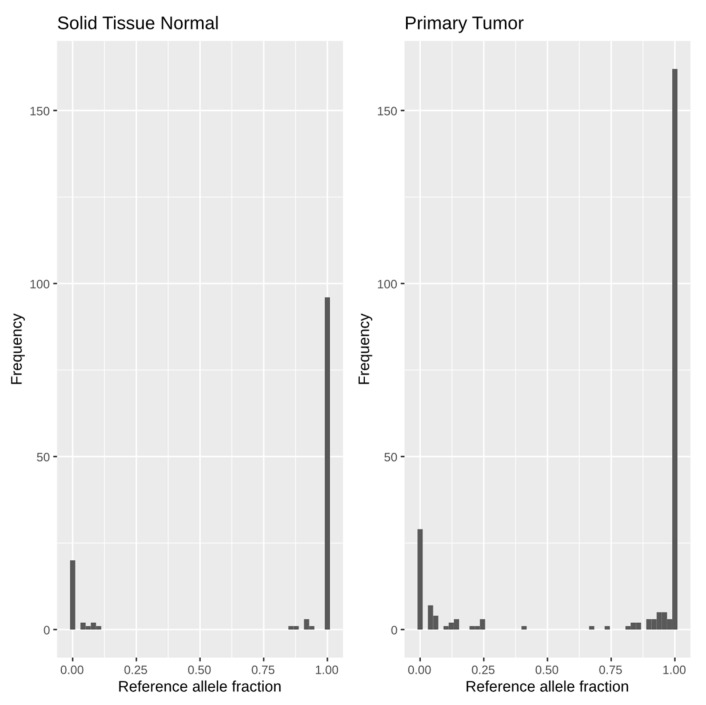
LOI of *HM13* in ccRCC. Allele fraction plots of normal solid tissue (left (solid tissue normal), N = 128) and stage 1 cancer samples (right (primary tumor), N = 240) demonstrate imprinting and LOI of *HM13* (rs6059874) in normal solid tissues resp. cancer. In these plots, the fraction of reference allele across all samples is plotted per SNP. With perfect imprinting, heterozygous samples either coincide with the homozygous reference allele (fraction of about 1) or homozygous alternative allele (fraction of about 0) samples. Hence, in normal solid tissue samples, the lack of a heterozygous peak indicates imprinting. Despite some minor loss-of-imprinting in normal solid tissue samples, there is a clear increase of LOI in ccRCC, indicated by a large increase of samples with allele fractions between 0 and 1.

To study imprinting deregulation as an early contributor to ccRCC development and progression, we evaluated LOI and differential expression (DE) of the putatively imprinted genes in stage 1 ccRCC versus normal solid tissues, identifying one gene featuring increased biallelic expression in cases associated with expression upregulation, compatible with canonical loss-of-imprinting: *HM13* (Table 1, Figure 1). *CRYBB2P1* shows signs of LOI as well (*p* = 6.33 × 10^−2^), yet we focused on *HM13*, as for *CRYBB2P1* cis-eQTL effects rather than imprinting have been reported in the literature (see below). For *HM13*, we verified that overexpression was (at least partially) driven by differential imprinting by first identifying LOI samples (see Section 2) and subsequently comparing the expression of LOI tumor with non-LOI tumor and matched solid tissue normal samples. *HM13* LOI samples featured *HM13* overexpression versus non-LOI (Log_2_FC = 0.37, *p* = 1.27 × 10^−2^) and solid tissue normal (Log_2_FC = 0.91, *p* = 3.00 × 10^−7^) samples, in line with canonical LOI. As all solid tissue normal samples were obtained as tumor-adjacent tissue from renal cancer patients, we also performed paired analysis between (non-)LOI ccRCC tumor samples and their matched normal solid tissues. Despite the very low number of ccRCC LOI samples with matched normal solid tissue, both analyses showed significant overexpression in cancer with the largest effect size for LOI: LOI vs. solid tissue normal: Log_2_FC = 1.09, *p* = 3.01 × 10^−3^, *n* = 5; non-LOI vs. solid tissue normal: Log_2_FC = 0.64, *p* = 1.00 × 10^−13^, *n* = 66.

Subsequently, we evaluated possible causes of LOI. We first considered DNA methylation at the *HM13* locus (Supplementary Table S1). Infinium HumanMethylation probe sites demonstrating approx. 50% methylation in solid normal tissue (indicative of imprinting regulatory sites) were not differentially methylated in stage 1 LOI tumor samples compared to stage 1 tumor samples without LOI or compared to solid normal tissue. When considering all probes, there was no differential methylation between stage 1 tumor patients with or without LOI either, except for a single probe set (cg21427119) (Supplementary Table S1). Among 10 probes that showed differential methylation between stage 1 tumor patients with LOI vs. STN, only a single probe set stood out; the site targeted by probe set cg21427119 (located in exon 4), with LOI samples showing significantly lower DNA methylation than STN (*p* = 1.34 × 10^−3^), while this is not the case in the overall tumor vs. STN comparison (*p* = 2.99 × 10^−1^, Supplementary Table S1). As *HM13* is located on the often gained 20q region [19], we also assessed the impact of copy number alterations. *HM13* copy number gains were present in 13.2% of stage 1 ccRCC tumors, and in this subset, copy number gain was positively associated with *HM13* expression (stage 1: Log_2_FC = 0.67, *p* = 2.80 × 10^−6^). On average, LOI samples featured more 20q copy number gain (29%, vs. 13.2% overall), yet this result was not statistically significant (chi-squared test, stage 1: *p* = 1.73 × 10^−1^). No *HM13* mutations were found in ccRCC stage 1.

**Figure 2:**
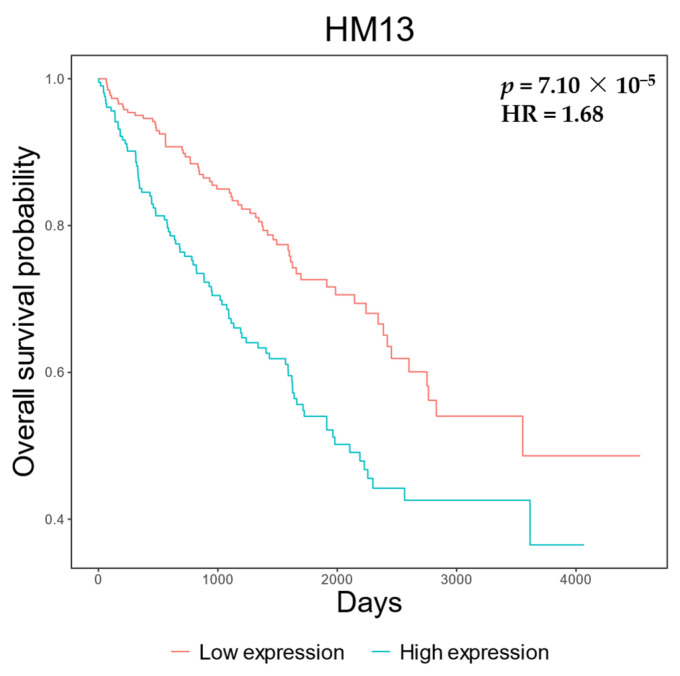
Overexpression of *HM13* leads to worse prognosis in ccRCC. Kaplan–Meier plot with *p*-value and hazard ratio obtained from Cox regression of *HM13* expression corrected for age and sex.

**Figure 3:**
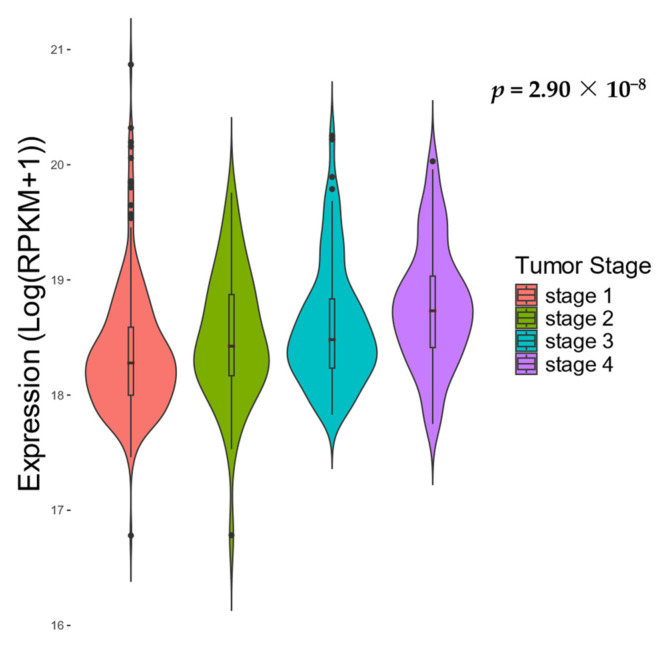
*HM13* overexpression with tumor stage. Violin plots show that *HM13* expression gradually increases with higher tumor stage.

Finally, we evaluated the clinical implications of overexpression. Cox regression (adjusted for age and sex) revealed that *HM13* overexpression was significantly associated with worse prognosis (*p* = 7.10 × 10^−5^, hazard ratio = 1.68, Kaplan–Meier plot in Figure 2). Strikingly, *HM13* expression increased as the tumor progressed throughout clinical stages (*p* = 2.90 × 10^−8^; Figure 3). Upon additional adjustment for tumor stage, the Cox regression results were no longer significant, though a similar observation of the hazard ratio persisted (*p* = 1.04 × 10^−1^, HR = 1.28). The same analysis of solely stage 1 tumors yielded no significant results (*p* = 0.44). Similarly, *HM13* survival results on the Human Protein Atlas website [20] indicate that overexpression in ccRCC does in fact lead to a clearly lower 5-year survival rate “https://www.proteinatlas.org/ENSG00000101294-HM13/pathology/renal+cancer/KIRC (accessed on 31 July 2024; *p* = 1.10 × 10^−7^, Supplementary Figure S1)”. 

Of interest, *IGF2* was not detected as imprinted as none of the *IGF2* SNPs passed our imprinting detection filters. More specifically, for the three SNPs with sufficiently high allele frequencies in the population, median imprinting estimates were below 0.8. Importantly, this indicates that this gene already features major LOI in normal solid tissue samples, in line with previous observations for healthy tissues [8,17]. We further evaluated whether there was more LOI in cases than in normal solid tissues, yet this was clearly not the case (adjusted *p*-value = 1). Cox regression (adjusted for age and sex) indicated that *IGF2* overexpression might be modestly associated with a worse prognosis, although the effect size is very limited (*p* = 1.03 × 10^−1^, HR = 1.06). However, in the case of *IGF2*, there is no association with tumor stage (ANOVA *p* = 0.60). Upon additional adjustment for tumor stage (*p* = 8.87 × 10^−2^, HR = 1.06) or only the analysis of stage 1 tumors (*p* = 3.37 × 10^−1^, HR = 1.06), the Cox regression shows a similar outcome. Results for *CRYBB2P1* were at first sight compatible with (loss-of-) imprinting in kidney cancer, yet we deem this result to be more likely to be caused by a cis-eQTL effect, possibly compounded by mismapping of reads between the *CRYBB2P1* pseudogene and its *CRYBB2* ancestral gene (see Section 4).

## 4. Discussion

Using a genome-wide approach, we identified about 20 genes to be putatively imprinted in normal solid tissue kidney. As the used methodology makes several assumptions [17], additional validation—preferably using parents and offspring trio data—would be required to be fully sure about the imprinting status. However, the large majority of the genes reported are known to be imprinted and/or are located in the immediate neighborhood of imprinting control regions. There is a substantial number of genes featuring significant LOI, yet typically without differential expression (e.g., Pterin-4 Alpha-Carbinolamine Dehydratase 2 (*PCBD2*)), or associated with expression downregulation (e.g., *MEST*, Zinc Finger Protein 331 (*ZNF331*)). The exact implications of such LOI remains unclear [17], and even false-positive results due to partial imprinting—where, e.g., normal cells featuring biallelic expression are present at increased fractions in tumor tissue—cannot be excluded. Full-length transcript single-cell RNA-seq will most likely be required to fully unravel the underlying molecular biology.

In ccRCC, *HM13* was the sole gene exhibiting both significant LOI and expression upregulation, i.e., canonical LOI. Canonical LOI of *HM13* was previously also demonstrated in breast cancer [17]. Particularly, *HM13* LOI samples feature overexpression, both compared to normal solid tissues and non-LOI cancer samples. Strikingly, *HM13* overexpression was clearly associated with tumor stage, and even—upon adjustment for sex and age—with survival. Yet, upon adjustment for tumor stage, the association of *HM13* expression with survival independent of clinicopathologic parameters could not be demonstrated. The previously demonstrated LOI of *HM13* in breast cancer [17] had unclear clinical implications. In lung cancer, including aberrant allelic *HM13* expression of cancer cells provided added value to a diagnostic grading model [21]. Also, functional experiments indicate clinical relevance by supporting an oncogenic impact of *HM13* overexpression. *HM13* expression has already been associated with prognosis in pan-cancer analysis [22,23], among which ccRCC is included [22]. Hence, this is the first study that clearly links these parts together and demonstrates that LOI is an important cause of overexpression of *HM13* in ccRCC, leading to worse patient survival outcomes.

In breast cancer, LOI was demonstrated to be associated with hypomethylation of putative imprinting-associated CpGs (~50% methylation) [17], yet we were not able to confirm this in stage 1 renal cancer. On the other hand, *HM13* is located on chromosome 20q, known to be commonly gained in cancer [19], and also leading to *HM13* expression upregulation in stage 1 ccRCC. Moreover, we demonstrated that ccRCC LOI is not—or at most limited—associated with copy number gain in stage 1. Hence, these results suggest that *HM13* LOI is relevant in cancer and may have multiple causes, including copy number gains and aberrant imprinting. Combined, these results argue for more in-depth functional pan-cancer analyses of *HM13*—a signal peptide peptidase involved in epitope generation—and the causes and impact of its deregulation in cancer.

Additionally, for *CRYBB2P1*, the results were compatible with (loss-of) imprinting. Previous research has shown that *CRYBB2P1* expression is increased in African American tumors compared to white American tumors in BRCA [24]. However, this required refined alignment of *CRYBB2P1* reads due to potential cross-mapping with the ancestral *CRYBB2* gene, which has also been attributed ethnic-specific disparity regarding cancer risk [24]. After further exploration of our findings, combined with the fact that *CRYBB2P1* has not yet been reported as imprinted, we deem our *CRYBB2P1* results more compatible with a tumor-specific cis-eQTL effect (possibly compounded by alignment artefacts) than with (loss-of) imprinting. When focusing on *IGF2*, we already found clear LOI in normal solid tissue kidney, even to the extent that *IGF2* SNPs did not meet our predetermined imprinting quality control filters. However, we did not find *IGF2* LOI to be more frequent in stage 1 ccRCC, contrasting previous results in ccRCC as well as other cancer types [7,8,9,10]. One potential explanation is that TCGA partially relies on apparently normal solid tissues from cancer patients, i.e., samples derived from tumor-adjacent tissue. Since *IGF2* LOI is already present in healthy tissues and increases cancer risk [9], it is possible that we detected too much cancer-associated LOI in “normal” samples to find significant differences in cancer. This is especially true because LOI has been found to occur early in carcinogenesis [6], which means that some of these tumor-adjacent tissues have started the oncogenic transformation, with LOI in *IGF2* as one of the early hallmarks. Note that this is a limitation inherent to our study, since no normal kidney samples from non-cancer patients were available.

In summary, here we demonstrated clear LOI of *HM13* in renal cancer causing *HM13* overexpression, which leads to poor prognosis.

## Figures and Tables

**Table 1:** LOI and DE results in ccRCC for imprinted genes identified in solid tissue normal kidney.

Gene	N	Î	p (LOI)	p (DE)	Log_2_FC
*ZDBF2*	6	0.98	4.05 × 10^−1^	9.90 × 10^−16^	−1.36
*COPG2IT1* ^$^	5	0.95	2.75 × 10^−1^	9.16 × 10^−25^	−1.65
*MEST*	2	0.9	6.30 × 10^−5^	1.05 × 10^−14^	−1.77
*H19*	2	0.93	1.89 × 10^−1^	8.68 × 10^−1^	0.05
*MEG3*	7	0.95	9.09 × 10^−1^	8.68 × 10^−1^	−0.05
*PWAR6* ^$^	4	0.99	3.71 × 10^−1^	4.84 × 10^−29^	−1.16
*SNHG14*	14	0.99	2.95 × 10^−1^	9.04 × 10^−12^	−0.73
*IPW* ^$^	2	0.98	4.82 × 10^−1^	1.36 × 10^−18^	−0.76
*ZNF597*	5	0.95	3.49 × 10^−1^	1.30 × 10^−2^	0.22
*PEG3*	6	0.97	1.59 × 10^−1^	9.90 × 10^−16^	−2.66
*ZNF331*	3	0.97	5.01 × 10^−25^	4.32 × 10^−6^	−1.12
*HM13*	3	0.97	2.74 × 10^−3^	9.90 × 10^−16^	0.60
*PCBD2*	2	0.95	3.25 × 10^−5^	6.53 × 10^−1^	0.04
*CRYBB2P1* **	2	0.82	6.33 × 10^−2^	2.02 × 10^−10^	0.98
*LLGL2* **	2	0.85	2.99 × 10^−1^	9.90 × 10^−16^	−1.35
*SLC9A3R2*	1	0.88	5.71 × 10^−8^	8.68 × 10^−1^	−0.02
*PEG10*	1	0.98	5.93 × 10^−1^	1.15 × 10^−4^	−0.52
*NDN*	1	0.99	9.71 × 10^−3^	5.03 × 10^−1^	0.10
*GNAS*	1	0.99	8.05 × 10^−1^	6.53 × 10^−1^	−0.05
*RPH3AL*	1	0.97	3.91 × 10^−12^	1.17 × 10^−2^	−0.29
*HLA–DQA2* ^$^*	2	0.82	5.64 × 10^−14^	1.82 × 10^−15^	1.56

Candidate imprinted genes with subsequent FDR-adjusted *p*-values assessing statistical significance of LOI (P (LOI)) and DE (P (DE)), as well as Log_2_FC (tumor/solid tissue normal) and mean estimated imprinting across SNPs (î). N denotes the number of SNPs demonstrating significant putative (5% FDR) imprinting. Only *HM13* is compatible with canonical LOI (no known RME, P LOI, and P DE < 0.05 and Log_2_FC > 0.5). ^$^ No gene-level expression data provided by Xena TCGA public data hub, differential expression was evaluated by combining *t*-tests of normalized SNP-level data. * Most likely to feature RME. ** Novel candidate imprinted genes.

## Data Availability

The results published here are based upon data generated by the TCGA Research Network “https://www.cancer.gov/tcga (accessed on 1 July 2020)” and are accessible upon application through the Genomic Data Commons Data Portal “https://portal.gdc.cancer.gov/ (accessed on 1 July 2020)”.

## Supplementary Materials

The following supporting information can be downloaded at: https://www.mdpi.com/article/10.3390/biom14080936/s1, Table S1: Differential methylation across *HM13* in KIRC, Figure S1: Kaplan-Meier graph from Human Protein Atlas for the *HM13* gene in KIRC.
