# Supplementary material for: Loss-of-Imprinting of HM13 Leads to Poor Prognosis in Clear Cell Renal Cell Carcinoma
Source: Biomolecules. 2024 Aug 2;14(8):936. doi: 10.3390/biom14080936 (PMC11352930; doi:10.3390/biom14080936)
Supplement: Supplementary file 1 [file biomolecules-14-00936-s001.zip › biomolecules-3059715-supplementary.pdf]

Supplementary Data

Supplementary Table S1. Differential methylation across *HM13* in ccRCC.

	STN (beta)	Tumor stage 1		Tumor all stages (beta)	Contrasts (adjusted p-value)		
		LOI (beta)	No LOI (beta)		Stage 1 LOI vs STN	Stage 1 LOI vs no LOI	All stages tumor vs STN
cg18471488	0.80341	0.671	0.6875	0.6387	3.18×10^{-15}	0.813	6.95×10^{-44}
cg04306926	0.92784	0.8858	0.9044	0.8887	3.54×10^{-5}	0.648	2.95×10^{-18}
cg12940420	0.89713	0.8362	0.8564	0.8485	3.56×10^{-5}	0.648	1.27×10^{-24}
cg22773914	0.01507	0.024	0.0197	0.0184	0.000134	0.648	6.74×10^{-11}
cg21427119	0.87946	0.7633	0.8777	0.8693	0.00134	0.146	0.299
cg24010885	0.00978	0.0122	0.014	0.0126	0.00305	0.648	1.02×10^{-14}
cg08511818	0.9094	0.8651	0.8771	0.8601	0.00362	0.682	9.75×10^{-19}
cg11478534	0.90824	0.8825	0.9013	0.9017	0.00617	0.611	0.00371
cg04803921	0.03959	0.0479	0.0438	0.0437	0.0109	0.648	1.15×10^{-5}
cg26833395	0.01226	0.0142	0.0154	0.0145	0.0126	0.648	1.19×10^{-10}
cg01412220	0.94167	0.9322	0.9425	0.9415	0.117	0.648	0.911
cg25935716	0.01139	0.0128	0.0129	0.0125	0.152	0.898	9.59×10^{-6}
cg04839274	0.01728	0.02	0.0219	0.0209	0.152	0.648	4.89×10^{-11}
cg14611750	0.08547	0.0954	0.0941	0.0936	0.324	0.898	4.28×10^{-5}
cg12576082	0.01891	0.022	0.026	0.0232	0.428	0.648	4.18×10^{-8}
cg00890916	0.87414	0.8875	0.9076	0.9105	0.428	0.648	1.36×10^{-21}
cg25645178	0.34297	0.3865	0.3548	0.3608	0.519	0.648	0.165
cg20129782	0.33373	0.308	0.3263	0.3223	0.597	0.682	0.293
cg17840843	0.31419	0.3468	0.3231	0.3253	0.605	0.648	0.34
cg25083781	0.05116	0.0538	0.0566	0.0552	0.605	0.648	3.75×10^{-5}
cg15815607	0.44519	0.472	0.4413	0.4477	0.65	0.648	0.848

cg24607140	0.55562	0.5746	0.5558	0.5626	0.65	0.648	0.475
cg19617948	0.44223	0.4671	0.444	0.4493	0.65	0.67	0.591
cg26038708	0.13012	0.1244	0.145	0.1386	0.65	0.648	0.00962
cg25359645	0.40493	0.4291	0.406	0.4127	0.65	0.648	0.561
cg02146091	0.43589	0.4607	0.4362	0.4428	0.65	0.648	0.591
cg06000530	0.41433	0.4305	0.4221	0.4243	0.748	0.866	0.475
cg15677825	0.07009	0.0712	0.0839	0.0805	0.814	0.611	$1.6x10^{-10}$
cg22013795	0.04736	0.0474	0.0515	0.0503	0.999	0.648	0.00676

First four columns show beta values for methylation of probes, last three columns show FDR adjusted p-values of contrasts. Tests shown are stage 1 tumor LOI samples vs solid tissue normal, stage 1 LOI tumor samples vs stage 1 no LOI tumor samples, and tumor vs solid tissue normal samples across all stages (full methylation dataset). STN: Solid Tissue Normal.

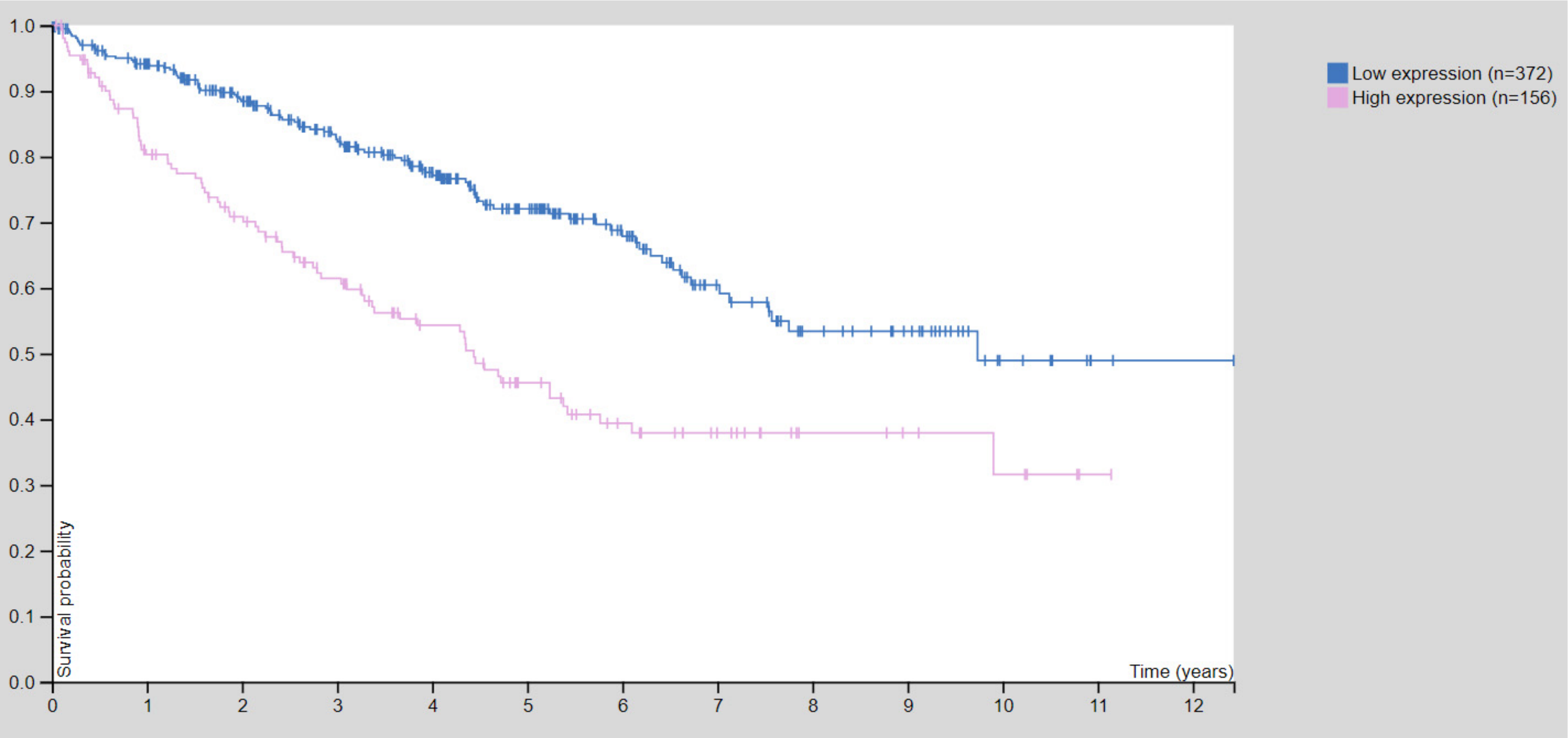

Supplementary Figure S1. Kaplan-Meier graph from Human Protein Atlas "https://www.proteinatlas.org/ENSG00000101294-HM13/pathology/renal+cancer/KIRC (accessed on 1 August 2024)" for the *HM13* gene in KIRC.
